# Reconstruction of sciatic nerve after traumatic injury in humans - factors influencing outcome as related to neurobiological knowledge from animal research

**DOI:** 10.1186/1749-7221-7-7

**Published:** 2012-10-10

**Authors:** Amanda Maripuu, Anders Björkman, Isabella M Björkman-Burtscher, Peter Mannfolk, Gert Andersson, Lars B Dahlin

**Affiliations:** 1 Departments of Hand Surgery, Lund University, Lund, Sweden; 2 Radiology, Lund University, Lund, Sweden; 3 Neurophysiology, Skåne University Hospital Malmö and Lund, Lund, Sweden; 4 BioImaging Center and Department of Clinical Sciences Malmö, Lund University, Lund, Sweden; 5 Department of Hand Surgery, Skåne University Hospital, SE-205 02, Malmö, Sweden

**Keywords:** Sciatic nerve injury, Nerve regeneration, Reconstruction, Outcome, fMRI, Dorsal root ganglia

## Abstract

**Background:**

The aim was to evaluate what can be learned from rat models when treating patients suffering from a sciatic nerve injury.

**Methods:**

Two patients with traumatic sciatic nerve injury are presented with examination of motor and sensory function with a five-year follow-up. Reconstruction of the nerve injury was performed on the second and third day, respectively, after injury using sural nerve grafts taken from the injured leg. The patients were examined during follow-up by electromyography (EMG), MRI and functionalMRI (fMRI) to evaluate nerve reinnervation, cell death in dorsal root ganglia (DRG) and cortical activation; factors that were related to clinical history in the patients.

**Results:**

One patient regained good motor function of the lower leg and foot, confirmed by EMG showing good activation in the leg muscles and some reinnervation in the foot muscles, as well as some sensory function of the sole of the foot. The other patient regained no motor (confirmed by EMG) or sensory function in the leg or foot. Factors most influential on outcome in two cases were type of injury, nerve gap length and particularly type of reconstruction. A difference in follow-up and rehabilitation likely also influence outcome. MRI did not show any differences in DRG size of injured side compared to the uninjured side. fMRI showed normal activation in the primary somatosensory cortex as a response to cutaneous stimulation of the normal foot. However, none of the two patients showed any activation in the primary somatosensory cortex following cutaneous stimulation of the injured foot.

**Conclusions:**

In decision making of nerve repair and reconstruction data from animal experiments can be translated to clinical practice and to predict outcome in patients, although such data should be interpreted with caution and linked to clinical experience. Rat models may be useful to identify and study factors that influence outcome after peripheral nerve repair and reconstruction; procedures that should be done correctly and with a competent team. However, some factors, such as cognitive capacity and coping, known to influence outcome following nerve repair, are difficult to study in animal models. Future research has to find and develop new paths and techniques to study changes in the central nervous system after nerve injury and develop strategies to utilize brain plasticity during the rehabilitation.

## Background

Traumatic sciatic nerve injuries are not unusual in the context of war, but they are rare in civilian healthcare compared to peripheral nerve injuries in the upper extremity. The incidence of peripheral nerve injuries in Sweden is 13.9 per 100000 inhabitants and year, out of which only 2% are injuries to the sciatic nerve at hip and thigh level [1]. The consequences of loss of sciatic nerve function are severe and despite improved microsurgical techniques functional outcome in patients often remains poor [2]. The neurological deficits seen after injuries to peripheral nerves hinder the patients, who often are young adults, in activities of daily living and at work and thus bring large costs to society [3,4]. Therefore, it is of great importance to improve existing treatment strategies and furthermore to develop new treatment strategies based on new knowledge in neurobiology [5]. Most animal research done on peripheral nerve repair and reconstruction use the rat sciatic nerve as a model for injury.

Here, an overview of the literature on factors influencing outcome after nerve repair and reconstruction [6,7] is presented followed by two cases of traumatic nerve injury that illustrate the factors influencing outcome after sciatic nerve reconstruction in humans. The clinical cases are related and compared to the knowledge gained from neurobiological research using the rat sciatic nerve injury model. The aim of the present case reports and review is to evaluate what can be learned from the rat sciatic nerve injury model when treating humans with such an injury, and furthermore to predict outcome in patients suffering from sciatic nerve injury. Here, we present two patients with traumatic sciatic nerve injury with different outcome of motor and sensory function with a five-year follow-up and related function to present knowledge about neurobiology after sciatic nerve injuries.

## Material and methods

This paper is divided into two parts. The first part is a literature review of factors influencing outcome after reconstructive surgery in peripheral nerves based on experimental and clinical studies. The factors were chosen from key references [2,6,7]. A search was conducted in the PubMed database for each factor. The key words “peripheral nerve injury and repair”, combined with each factor or key words for each factor, gave several articles for each factor as presented (Table 1). The limit “English” was used consistently. In two instances when the search generated a large quantity of articles the limit “review” was added. Articles were then chosen based on relevance to the aim presented above with focus on studies and reviews concerning sciatic nerve injuries and outcomes, such as progress of regeneration and result after reinnervation. Some articles referred to original articles that had been missed in the first database search. These were included as well.

**Table 1:** Description of search strategy in the PubMed database

**Search terms**	**Limits**	**Result**
Peripheral nerve injury signal transduction	English, review.	**159**
Peripheral nerve injury repair cell death	English.	**76**
Peripheral nerve injury repair delay	English.	**76**
Peripheral nerve injury repair age	English, review.	**17**
Peripheral nerve injury repair level sciatic	English.	**34**
Peripheral nerve pre-degeneration	English.	**25**
Sural nerve donor-site morbidity	English.	**26**
Peripheral nerve misdirection	English.	**64**
Peripheral nerve injury repair plasticity	English.	**87**

The second part is constituted of a description of two patients treated at the Department of Hand Surgery, Skåne University Hospital in Malmö, Sweden.

All experimental data reported and conducted by the authors were performed with approval of the appropriate ethics committee (Lund University; several reference numbers; provided by request). In addition, the ethics committee (humans) approved the follow up procedures (Lund University; reference number on request) and were performed according to the declaration of Helsinki. Both patients gave their consent for the report to be published.

After injury to a peripheral nerve, several intracellular signaling pathways, initiated at the site of the lesion, convey information of the event to the neuron cell body. As a result of these signals, the cell can either go into regeneration mode or enter a pathway to programmed cell death, i.e. apoptosis. Similar alterations in signal transduction pathways also occur in Schwann cells (SC). Successful nerve regeneration depends on Schwann cell activation and proliferation as well as changes in the neurons themselves [8]. When the axon is divided, Ca^2+^-ions flood into the cell causing the cell membrane to reseal. The influx of ions also creates an action potential that constitutes the first signal of injury. The normal retrograde transport of signaling molecules, such as nerve growth factor (NGF), from the periphery to the cell body is inhibited and this in itself a signal (i.e. negative signal), which alerts the neuron that an injury has occurred [8]. Growth factors, such as leukemia inhibitory factor (LIF) and ciliary neurotrophic factor (CNTF), present at the site of injury, bind to a tyrosine kinase receptor on the nerve cell. Thus, a signaling cascade (i.e. positive signal) is initiated, where phosphorylation and activation of subsequent enzymes end in activation of transcription factors [9]. The transcription factors extracellular signal-regulated kinase 1/2 (ERK 1/2), c-Jun N-terminal kinase (JNK), activating transcription factor 2 (ATF2) and signal transducer and activator of transcription 3 (STAT3) are activated at the site of injury and then transported by motor proteins along microtubules to the nucleus where they are imported by means of nuclear localization signals [9,10].

At the tip of the regenerating axon a growth cone with fingerlike filopodia and veil-like lamellipodia is formed. The growth cone interacts with the environment through surface integrins [8]. There are both attracting and repulsive signals acting on cytoskeleton elements. Direction of the growth cone is achieved by polymerization or destruction of actin filaments as well as protrusion of microtubules in the growth cone [11].

The SC, myelinating or non-myelinating, act as a supportive cell to the neuron and has a close contact with the outgrowing axons [12]. When the axon is injured, signaling pathways similar to those in the neuron are present in the SC as well, causing it to shed its myelin and start proliferating. A multitude of genes are up-regulated as well as down-regulated in response to e.g. ERK1/2, which is activated shortly after injury [8]. A time follows where the purpose of the SC is to ensure a favorable milieu for growing axons, including preparing the basal lamina with an encouraging surface for the outgrowing axons. Positive growth factors are released and the proliferating cells constitute the bands of Büngner, which act as guides for the regenerating axons [13]. When regeneration is complete the SCs take up their former role as a provider of neurotrophins, like NGF and glial cell-derived neurotrophic factor (GDNF). The close one-to-one contact between neuron and glial cell is reinstated [5]. The type of axon will determine whether the SCs produce myelin or not by contact through e.g. the neural cell adhesion molecule (N-CAM) [14].

### Cell death

Damage to the axon may lead to death of the neuron, impairing the possibilities for functional recovery [5]. In addition, SCs also go through apoptosis at the site of the lesion and in the distal nerve segment [15]. There are two intracellular pathways leading up to apoptosis; the intrinsic pathway, where proapoptotic enzymes are released from the mitochondria, and the extrinsic pathway, where the cell reacts to activation by receptors binding to cell surface death receptors [8]. In young animals, cell death is more common; probably due to the natural part apoptosis takes in neural development [16]. In studies with young animals, enzymes called caspases play a major role for the development of apoptosis. This has not been seen in adult motor and sensory neurons, but in SCs, and in satellite cells surrounding sensory neurons in dorsal root ganglia; thus, caspase 3 may be a reliable marker of apoptosis in such cells [17]. Sensory neurons are more susceptible to proapoptotic signals, which can be illustrated by the loss of dorsal root ganglia (DRG) mass seen in rats following a nerve injury [18]. There are studies in the rat model where magnetic resonance imaging (MRI) is used to evaluate the size of the DRG at the level corresponding to the peripheral nerve injury [18]. This may also be used to illustrate cell death in patients.

### Age

Age of the injured individual is one of the most recognized factors determining outcome after reconstruction [19]. Children generally are considered to have a better outcome after peripheral nerve injuries; an advantage that is most notable before the age of 10 with a decline in outcome in the late teens [6,20]. Several possible explanations for this exist. The shorter distance between injury and target is one factor. Another factor is the greater capabilities of the young brain to adjust, i.e. plasticity, to the altered nerve signal pattern from the periphery through the injured nerve induced by misdirected growth of particularly the axons of the sensory neurons [19]. As we will see later, plasticity is an important factor for outcome after nerve repair and reconstruction [21]. Children have a greater learning capacity in general, for example learning languages, and it is believed that this skill complies with learning to cope with a changed sensibility as well [20].

### Timing of nerve repair and reconstruction

The optimal time for repair and reconstruction of transected or lacerated nerve trunks is frequently discussed. Out of necessity, repair and reconstruction has often been delayed because of unfavorable wound conditions and the risk for infection. However, repair and reconstruction of closed nerve injuries with no apparent regain of function may also be delayed [2]. New neurobiological data, and also clinical observations, indicate that early nerve repair and reconstruction promotes axonal outgrowth and final recovery in the patient [22]. Neuronal cell death is more frequent after delayed repair, and more pronounced in sensory neurons than in motor neurons [23]. The neurons also loose regenerative potential, which is illustrated by the decreased expression of activating transcription factor 3 (ATF-3), a retrograde signal involved in inducing the genetic growth program, after delayed repair [24].

SCs distally to an injury react rapidly to denervation with de-differentiation and proliferation or apoptosis [8]. While proliferation is necessary to support axonal outgrowth, apoptosis of SCs increase with time; thus, the longer delay the less possibility of neuron regeneration [15,17]. Remaining SCs also loose their ability to react to axonal signals after prolonged denervation [12]. Furthermore, there is a greater number of non-myelinating SCs along the distal segment with delayed nerve repair [24].

Timing in cases with closed injuries is a separate matter, where the difficulty lies in determining which injuries should be explored. Overall, a three months limit is suggested, during which the clinical progress should be monitored by repeated clinical examinations, i.e. active surveillance, and in some specific cases – EMG investigation [25]. If no return of motor function can be seen at three months exploration should be considered. Exploration of a closed nerve injury with insufficient recovery is also an alternative, where the condition of the nerve and the extent of the injury can be tested intraoperative with nerve stimulator to judge the nature of any possible repair or reconstruction procedure [26]. However, in open injuries there is no reason to delay exploration and repair longer than absolutely necessary since the setting for regeneration is as best within the first few days following injury, after which the degree of activation in neurons and Schwann cells rapidly declines [24].

### Type and level of injury

The type and level of a nerve injury influence the result after repair and reconstruction in several ways [21]. There are several types of peripheral nerve injuries ranging from mild acute compression injuries which will resolve without treatment (if compression is relieved), through chronic compression injuries and compression injuries with damage to the axons to transection, laceration or even avulsion of a nerve root from the spinal cord [21]. In the case of transections and lacerations, where the whole nerve structure is divided, regeneration is difficult, if not impossible, unless surgical co-aptation of the nerve ends is performed.

The level of injury is important as related to time until target reinnervation and thus preservation of the target with a possibility to recover its function. A muscle without innervation will start to atrophy [27]. This starts within the first three months after injury [27,28] and the process reaches a critical level after two years [29]. Muscle atrophy is mainly non-reversible if such a critical time point is reached, and hinders reinnervation [6]. Sciatic nerve injuries at the level of the gluteal muscles have a worse outcome than injuries at the thigh level, probably due to the greater distance to target when the injury site is proximal in the leg [3,30]. There is also a larger amount of neuronal cell death with proximal injuries, i.e. injuries closer to the neuron cell body [5].

### Reconstruction technique

Transection or laceration of a nerve trunk will leave the proximal and distal nerve ends separated from each other. In these cases, it is important to establish continuity in the nerve in order for the axons to find their way over the area of scar tissue [25]. Direct repair is preferable, but not always possible, especially since tension in the repair has a negative influence on the result [31]. If direct repair is possible the nerve ends should be prepared by removal of necrotic tissue; then, approximated so that the fascicular pattern matches. Finally, the nerve ends are kept in position by tissue glue or sutures [32]. In order to bridge the gap in cases where direct repair is not possible a graft is needed. The current standard method is autologous nerve grafting with a dispensable sensory nerve. Multiple segments are placed side by side, without tension (preferably length >10% longer than gap due to shrinkage), to match the width of the nerve [32].

Some experimental data indicate that a motor nerve graft would be preferential in repair of motor nerves, but the supply of redundant motor nerves is limited leaving repair with a sensory nerve as standard [33,34]. In addition, the usefulness of a motor nerve graft, as compared to the gold standard sensory nerve graft, has not been shown in clinical cases.

Sacrificing a sensory nerve is not optimal and other alternatives are being investigated. Nerve conduits are synthetic or biological tubes, used instead of autografts, which have been tried and found useful, albeit only for short gaps [25]. Allografts have been used and work relatively well, but the need for immunomodulative treatment limits its use [25,35,36]. However, extracted nerve allografts, i.e. cellular content and myelin extracted, are available [37] and commercially obtainable in some countries. End-to-side repair is a much-studied technique, where the distal end of the transected nerve is sewn on to the side of a healthy nerve [32]. However, the technique is probably only suitable for a limited number of nerve injuries, such as injuries in the brachial plexus [38].

Nerve transfer is a technique where a nerve branch or some nerve fascicles close to the target is cut and sewn onto the distal end of the injured nerve [25]. It is an alternative that may be useful when the site of injury is far from the target and reinnervation is unlikely before muscle atrophy reaches a critical level. A less important nerve close to the target is then used and connected with the distal segment of the injured nerve. The technique may still suffer the problem of sacrificing another nerve [29]. Furthermore, the method relays on cerebral plasticity in order to execute the new function. However, several favourable techniques have been described, like the Oberlin procedure [39].

### Pre-degeneration

An injury to a nerve starts the complex process of degeneration in the distal segment as described above. Based on the fact that this process enhances growth of axons through a graft or the distal segment toward its target organ, the concept of pre-degeneration of nerve grafts was introduced. This means that the graft used for repair of the injured nerve trunk is damaged by crush or transection before use in reconstruction, and through this is already activated. Pre-degeneration has a positive effect on regeneration after nerve reconstruction [40]. The greatest effect is that of shortening delay of onset of axonal outgrowth, which is most evident between 3 and 14 days after the initial injury to the graft [41].

### Donor-site morbidity

The nerve most commonly used as a donor for autologous nerve transplantation is the sural nerve; i.e. a sensory nerve of the lower leg. This is due to its length, few branches and the relatively small consequences of its loss [32]. The area innervated by the sural nerve is located on the lateral aspect of the foot and heel. Although most patients are satisfied with donor site result [42], there often remains an area of anesthesia on the foot when further recovery is impossible [43]. Normally, this causes little discomfort. However, it is worth to note that recovery of sensation in this area is slower or less probable in a sciatic nerve injured patient, where the donor-site is ipsilateral to the injury [43].

### Type of nerve

The type of nerve injured accounts for differences in the inert regeneration potential between different peripheral nerves [6]. Pure motor nerves have the best regeneration potential. As mentioned above sensory neurons are more sensitive to injury, and many will not survive axonal transection [18]. However, even pure sensory nerves have an advantage in comparison with mixed nerves, which have both sensory and motor components. The different types of axons in mixed nerve trunks are normally organized in fascicles, but when the nerve is reconstructed the axons may find their way through the wrong endoneurial tubes. This gives rise to misdirection, a concept described further below. Different mixed nerves have different outcomes in motor function [6]. In sciatic nerve injuries the gastrocnemius muscle, innervated by the tibial branch of the sciatic nerve, often recovers well, while the anterior tibial muscle, innervated by the peroneal branch of the sciatic nerve and needed to extend the foot, often proves more difficult to restore [30]. One may consider the possibility that the potential of the axons to grow is better in the tibial nerve than in the peroneal nerve, which may have several causes. There is also a possibility that a coordinated input is needed to activate the elongated anterior tibial muscle [3].

### Misdirection

The mechanisms of how the regenerating axon finds its way back to the correct target are under discussion. Some studies indicate that there is preferential targeting, while others claim that axons innervating the wrong target are sorted out [5]. However, although many axons find the correct target, some do not. This affect the functional outcome after nerve repair and reconstruction [44]. Motor axons may end up connecting with other muscle fibers than originally, as can sensory axons after reinnervation supply another skin area than they originally did [45]. In reinnervation of muscle, one axon can innervate a greater number of muscle fibers than before. This leads to bigger motor units, which can be seen as large motor unit action potentials in EMG [46]. There is also an amount of polyinnervation, where one muscle fiber is activated by two or more axons, which may resolve with passing time [47]. In the rat sciatic nerve injury model, misdirection leading to simultaneous activation of antagonistic muscles leads to impaired gait [44]. In adult humans, motor function is less disturbed by misdirection than sensory function. As mentioned above, misdirection of sensory axons give rise to a changed signal pattern from the peripheral nerve to the brain requiring a learning process [20]. There is also a misdirection between axons from sensory and motor neurons, which will contribute to the disturbed function [7].

### Changes in the CNS

A peripheral nerve injury and reinnervation result in changes in the central nervous system both at the spinal and cerebral levels [45]. Both in the sensory and in the motor systems in the brain changes arise in two phases, first as a response to denervation and second in response to reinnervation of target organs. In the sensory system, the first phase consists of the removal of input, deafferentation, leading to expansion of the surrounding cortical areas. The first phase is followed by a period of reinnervation of target tissues and renewed sensory input [7]. This sensory input is changed, due to misdirection of the outgrowing nerve, resulting in a changed organization of the primary sensory cortex [48]. In the motor system loss of target muscle leads to loss of activity in the corresponding areas of the motor cortex. This is reversed with reinnervation [49]. The dynamics of the cerebral changes following a peripheral nerve injury can be studied using different neuroimaging techniques, such as functional magnetic resonance imaging (fMRI).

### Cognitive brain capacity

Although much variance in result after peripheral nerve repair and reconstruction can be attributed to the above mentioned factors, this does not account for the whole spectrum of patient outcome. Since rehabilitation is a learning process, part of the variance in clinical outcome may lie in the cognitive capacities of the injured individual [7]. It has been shown that certain abilities, such as verbal capacity and visuo-spatial logic ability, relates to a better functional sensibility following nerve repair [19]. Sensory training should be adjusted to the individual capacities and stage in the nerve reinnervation process [7]. Rehabilitation of motor function depends on several factors, such as motivation, misdirection, timing, and loss of muscle mass. The motivation and coping abilities of the patient are important to keep up with the sensory and motor training needed to achieve an acceptable outcome after peripheral nerve injury and repair [32].

## Case reports

### Case 1

**Figure 1:**
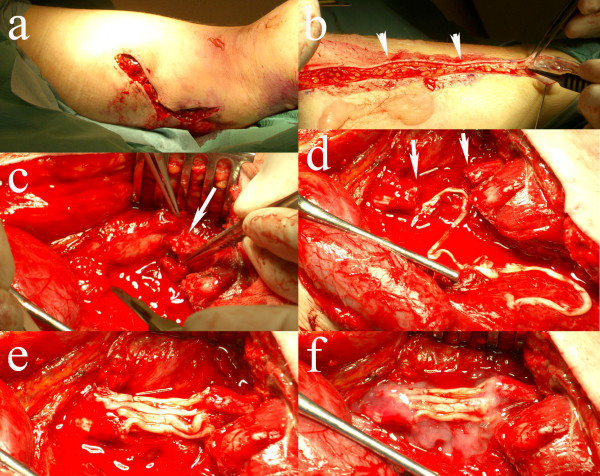
**Peroperative photos from case 1.a**) Wound of the patient on posterior part of thigh. **b**) Harvest of sural nerve graft (arrowheads) from the same lower leg. **c**) Injured sciatic nerve (arrow indicates distal nerve end). **d**) The injured sciatic nerve ends (arrows) with the long sural nerve graft. **e**) Eight segments of sural nerve graft attached with single sutures between the proximal (left) and distal (right) nerve ends. **f**) The sural nerve grafts glued with tissue glue (Tissel®).

A 26-year old man accidentally had a cut from a circular saw in the medial, posterior part of the right thigh during work. Due to vascular damage of the femoral vessels he suffered substantial blood loss and when he was brought to the emergency room he was in shock but awake. The damage to the femoral artery and vein was repaired immediately and circulation of the leg and foot was restored within three hours of injury. On examination the day after surgery it was discovered that the patient had loss of sensory and motor function matching the area of the sciatic nerve below the point of injury. On the third postoperative day the area was explored and the sciatic nerve was found to be transected. After trimming of the nerve ends there was a gap of 3–4 cm. The individual tibial and peroneal groups of fascicles could be identified in the wound and by electrical stimulation of the distal nerve end. The sural nerve of the injured leg was harvested and divided into eight segments, which were then used as grafts, 5 segments for the tibial component and 3 segments for the peroneal component (Figure 1). The grafts were applied with extended knee position and fixed with single 9–0 sutures and tissue glue (Tissel®).

The leg was immobilized in semi flexion for four weeks. The initial rehabilitation was without complication. Follow-up was conducted at our department, every three months the first year and then every six months. The patient used an orthosis during daytime for support and to hold the foot up and physical therapy to counter contractions. Sensory re-education was performed. He later had some tendency towards plantar flexion contracture, especially in the mornings, which was treated with an orthosis during night.

The physical therapy aimed to encourage counter contractions in the leg. Motor function progress was measured according to the British Medical Research Council (MRC) scale showing a continuous improvement of muscle force (Table 2) At final follow up (58 months) he, above the muscle force described in Table 2, also had M4 in toe extensor muscles and even M3+ in toe flexor muscles, although more concentration was required by the patient to activate the latter muscles. Regeneration was followed with Tinel’s sign. Sensory function was examined with Semmes-Weinstein monofilament conducted by an independent occupational therapist. At 24 months follow-up he could feel the 4.56 evaluator filament (indicating diminished protective sensation) on the mid lateral part of the sole of the foot. Apart from that he only had deep pressure sensation in the foot. He had no sensation over the heel. At 40 months follow-up he could feel the 4.31 evaluator filament (diminished light touch) in the mid lateral part of the sole of the foot and under the third and fifth toe. Under the big toe and the heel he could feel the 6.65 evaluator filament (deep pressure sensation) and in the rest of the sole of the foot he could feel the 4.56 evaluator filament (diminished protective sensation). At 58 months he could feel 3.61 evaluator filament in all toes and in most of the sole of the foot, except at the heel (4.31 evaluator filament). At no point did he have problems with cold sensitivity. He had some problems with allodynia, which was treated with tramadol hydrochloride (Tramadol®) for pain relief. An electromyography (EMG) done at 41 months showed decreased nerve conduction over knee level compared to the un-injured leg. In the majority of the muscles of the lower leg denervation activity was seen, most prominent in the distal muscles. However, in the gastronemius, long peroneal and anterior tibial muscles there were good voluntary activations. A low voltage response from the abductor hallucis muscle on stimulation of the tibial nerve at the ankle indicated that there was some reinnervation of the muscles of the foot.

**Table 2:** Gain of motor function related to time after surgery in case 1

**Time**	**Gastrocnemius M**	**Peronei Mm**	**Anterior tibial M**	**EHL**
**3 months**	0/5	0/5	0/5	0/5
**6 months**	0/5	0/5	0/5	0/5
**10 months**	4-/5	0/5	0/5	0/5
**17 months**	4/5	2/5	0/5	0/5
**23 months**	4+/5	4/5	3-/5	0/5
**29 months**	5/5	4/5	3+(4-)/5	1/5
**35 months**	5/5	4/5	4-/5	3/5
**42 months**	4+/5	4/5	4/5	4-/5
**58 months**	4+/5	4+/5	4/5	4/5

*M* muscle, *Mm* muscles, *EHL* extensor hallucis longus muscle. Values are Medical Research Concil (MRC) scale (0 = no contraction and 5 = full power).

### Case 2

A 16-year old man sustained an open fracture of the left femur after a motorcycle accident. The distal fracture segment perforated the posterior aspect of the thigh, thus lacerating the sciatic nerve proximally of the bifurcation of the peroneal and tibial nerve components, resulting in a gap between the nerve ends of 6–7 cm. The femoral blood vessels were intact. The fracture was treated surgically by insertion of a femur rod. The wound was revisited on the second postoperative day, considered to be sufficiently clean without necrosis to proceed to nerve reconstruction, and the nerve injury was repaired with autologous nerve grafts. The ipsilateral sural nerve was used as a donor for nerve grafting. A two-segment sural nerve graft was used to traverse the gap (maintenance unknown, probably were sutures used). The wound was infected postoperatively, where bacterial culture showed Bacillus cereus and the patient received treatment with clindamycin (Dalacin®). The wound then healed uneventfully. The leg was not immobilized post operatively. He was fitted with a foot-drop brace. Follow-up was conducted at an orthopedic clinic. He was reexamined with radiography to follow the healing of the femur fracture every six weeks during the first three months and then every six months. After 17 months the femur rod was removed due to pain. He had problems with pain during the first six months, which was treated with pregabalin (Lyrica®) with sufficient effect.

After 20 months there was little progress of nerve regeneration. An EMG was performed that showed denervation activity, fibrillations and positive sharp-waves in all the muscles of the lower leg, below the site of lesion. No voluntary units could be seen. In addition, no reaction in the tibial muscle after stimulation of the peroneal nerve at the knee was seen. Thus, there were no neurophysiologic signs of reinnervation of the lower leg. This related to the result of examination at our department 29 months after repair. At this point there was extensive atrophy of the muscles of the lower leg. Tinel’s sign was positive at a point 18 cm proximal to the medial malleolus, but without any detectable subjective or objective signs of sensibility in the lower leg or foot. No function, i.e. no voluntary contraction, in the muscles of the lower leg below the site of the lesion could be seen (Table 3). Different additional surgical procedures, like nerve transfers as palliation in the lower leg, were also considered [50] and discussed with the patient, but such procedures were declined.

**Table 3:** Gain of motor function related to time after surgery in case 2

**Time**	**Gastrocnemius M**	**Peronei Mm**	**Anterior tibial M**	**EHL**
**29 months**	0/5	0/5	0/5	0/5
**34 months**	0/5	0/5	0/5	0/5
**43 months**	0/5	0/5	0/5	0/5

*M* muscle, *Mm* muscles, *EHL* extensor hallucis longus muscle. Values are Medical Research Concil (MRC) scale (0 = no contraction and 5 = full power).

### MRI investigations

MRI data were acquired using a 3 T MRI system (Siemens Skyra, Erlangen, Germany).

#### fMRI and brain morphology

Functional data were acquired using a 32 channel head coil. During functional acquisition tactile stimuli of the sole of the right and left foot, respectively, were applied simultaneously with 3 pneumatic pads placed over the distal phalanx of first and second toe and over the distal part of the first metatarsal bone. Tactile stimuli were applied in a block design, alternating between individual stimulation of each foot, separated by a rest condition of no stimuli (e.g. right foot – rest – left foot – rest). All activation/rest block lengths were 17.5 s. Sensory stimulation was delivered using a pneumatically driven and electronically controlled stimulus system (pulse frequency = 1 Hz, pulse width = 100 ms, pressure = 2.5 bars) [51,52]. For functional imaging, a gradient-echo echoplanar imaging (GE-EPI) pulse sequence was used with scan parameters, TR/TE = 2500/30 ms, voxel size = 2×2×2 mm^3^, 33 slices and 112 dynamic scans. A high-resolution image volume was also acquired, using an anatomical 3D magnetization prepared rapid acquisition pulse sequence (MP-RAGE), with scan parameters TR/TE = 1900/2.54 ms, voxel size = 1×1×1 mm^3^ and 176 slices.

Prior to analysis, the functional image data were realigned, slice time corrected, co-registered to the anatomical MP-RAGE volume, and smoothed using a Gaussian kernel with FWHM = 5 mm. Statistical parametric maps were created using the general linear model (GLM). Four specific contrasts were evaluated for both individuals: (1) healthy foot > rest, (2) nerve injured foot > rest, (3) healthy foot > nerve injured foot and (4) nerve injured foot > healthy foot. All preprocessing and statistical analysis was performed using SPM8 [ http://www.fil.ion.ucl.ac.uk/spm].

#### Spinal MR

MR of the lumbar and sacral spine was acquired with the spine coil and a sagital 3D T2 SPACE sequence (Sampling Perfection with Application optimized Contrast using different flip angle Evolution) with voxel size = 0.6×0.6×0.6 mm^3^, 120 slices, TR/TE = 1500/136 ms, allowing image reconstruction in any plane. The cross sectional area of the dorsal root ganglion was measured on a reconstructed image through the largest portion of the ganglion and perpendicular to the length axis of the spinal nerve. Measurements were performed bilaterally for the fourth and fifth lumbar nerve (L4 and L5 nerve) and the first and second sacral nerve (S1 and S2 nerve).

## Results

### fMRI and brain morphology

No brain pathology was delineated on morphological images.

**Figure 2:**
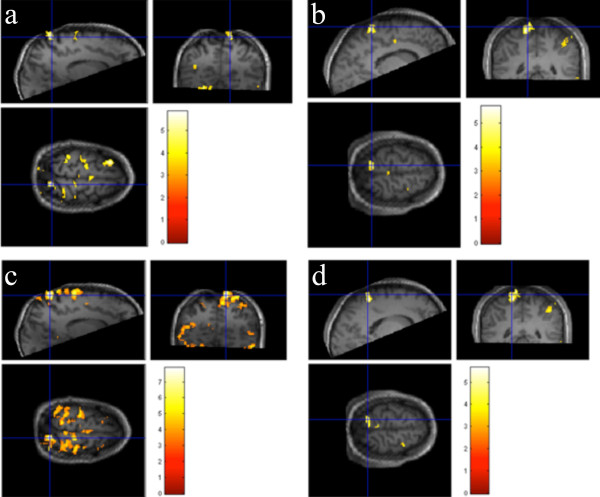
Activation t-maps, overlaid on the high-resolution MP-RAGE volume (threshold at t = 3.17, corresponding to p = 0.001, cluster size threshold = 10) for the contrast healthy foot > rest for the patients with right (patient 1) (a) and left (patient 2) (b) side sciatic nerve injury, respectively, and for the contrast healthy foot > nerve injured foot for the same two patients (c and d).

Activation maps were thresholded at p = 0.001 (uncorrected for multiple comparisons, corresponding to t = 3.17), and an additional cluster size threshold of 10 was applied. No activation was seen during stimulation of the foot ipsilateral to the sciatic nerve injury (nerve injured foot) while stimulation of the contralateral foot (healthy foot) resulted in activation in the sensory cortex (Figure 2a and b show activation t-maps for the contrast healthy foot > rest for both patients). Figure 2c and d show activation t-maps for the contrast healthy foot > nerve injured foot for both patients showing a significant activation difference between the healthy and the nerve-injured foot.

### Spinal MR

**Figure 3:**
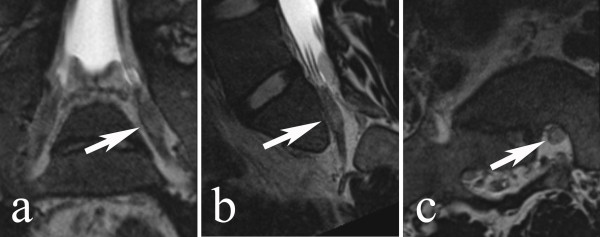
**Magnetic resonance images (T2 weighed CCI SPACE sequence) of the left S1 spinal nerve at the level of the dorsal root ganglion (arrows).** 1. Coronal view, 2. sagittal view and 3. axial view. All views are reconstructed projections parallel (coronal and sagittal) or perpendicular (axial) to the length axis of the nerve.

The sciatic nerve originates in the lumbar and sacral spinal cord (L4 to S3) and supplies motor and sensory innervation to the lower extremity (Figure 3). Maximum cross sectional areas of the dorsal root ganglia are given in Table 4. Cross sectional areas did not correlate to side of injury.

**Table 4:** **Cross sectional areas of the dorsal root ganglia in mm**^**2**^

**Nerve**	**Case 1**		**Case 2**	
	**Right (injured)**	**Left (healthy)**	**Right (healthy)**	**Left (injured)**
**L4**	**23.0**	**21.5**	**38.5**	**41.5**
**L5**	**19.5**	**24.5**	**36.5**	**44.0**
**S1**	**24.0**	**25.5**	**43.0**	**43.0**
**S2**	**11.5**	**13.5**	**21.5**	**20.0**

## Discussion

The two cases presented above had similar sciatic nerve injuries, but different outcome after the nerve reconstruction. The explanation may be found by examining the factors known to influence outcome after peripheral nerve injuries. The gap length has some influence, since a longer gap leads to a worse outcome. This has likely contributed to the poor result for the second case, which had a final gap of 11 cm. On the other hand, and more importantly, the manner of reconstruction differed between the cases as well. Even though they both received sural nerve autografts from the injured leg, the first patient was operated on using an eight-nerve segment reconstruction, while the second patient only got a two-segment graft. Due to the size ratio between the nerve and the segment graft, such a difference will most probably have implications on the efficiency of the axonal outgrowth through the graft and thus over the defect. A sufficient diameter of the graft is needed to attract a sufficient number of axons and to direct the axons on their right path [32]. The sciatic nerve is a thick mixed nerve and the individual sural grafts are very thin. The reconstruction performed on the second patient appeared to be insufficient, based both on the lack of reinnervation and on the clinical signs of severely impaired axonal outgrowth with only a Tinel’s sign 18 cm proximal to the medial malleolus. A neuroma forms mainly under conditions that prevent the regenerating axons to reach a target organ, these conditions being scar tissue or lack of guidance over a gap. One should stress that an appropriate number of segments provide the outgrowing axons with growth stimulating factors through a sufficient number of proliferating Schwann cells. No information is available on why not the contralateral nerve as well as the entire sural nerve was utilized in the second case to create a better reconstruction. Most probably, nerve regeneration should have proceeded better if multiple nerve graft segments have bridged the defect in the second case. Interestingly, experimental data have shown that axonal outgrowth is better in the larger tibial nerve than in the smaller peroneal nerve branch. This indicates that the amount of proliferating Schwann cells in the distal nerve segment contributes to attract outgrowing axons [53]. Thus, in cases where there is a shortage of nerve graft segments, the available segments should be directed to the tibial nerve due to its better regeneration capacity and a palliative tendon transfer could have compensated the lack of peroneal nerve function. Alternatives, such as extracted acellular nerve grafts [54], were not available in our country at the time of the injury and such grafts, although they lack Schwann cells and may be insufficient for the present nerve defect in the second case, could have contributed to axonal outgrowth. In addition, nerve transfers, as an alternative or a palliation procedure, may also be a possibility in the second case [50], but was declined for several reasons (e.g. no suitable nerve transfer in relation to a possible improvement and good outcome at the time when the patient was referred and opinion by the patient).

Other differences between the two cases were the method of follow up and the postoperative care, including the treatment of the limited local infection, given. The first patient was followed by a team used to this type of injuries and examined closely for signs of regeneration, and when such signs were found specific rehabilitation efforts were directed at the muscle or skin area recently reinnervated. A team that focused on the healing of the femur fracture followed the second patient. Subsequently, attention was directed against fracture healing and not towards the status of the regenerating nerve. The result was a delay in diagnosing the lack of regeneration until two years after the injury when a great part of the muscle mass of the lower leg was already lost and little hope of regaining function remained. If there are sparse signs of regeneration after the reconstruction of the sciatic nerve injury, like no advancement of Tinel´s sign and reinnervation of muscles, one should consider re-exploration of the injured area, particularly focusing on the distal coaptation, within an appropriate time perspective, as timing is crucial. Unfortunately, this was not done in the second case. Thus, active surveillance of nerve repairs and reconstructions is essential to follow nerve regeneration after repair or reconstruction procedures and to decide if a nerve injury should be re-explored, particularly if no advancement of Tinel´s sign is observed. The different treatment regimes may also have induced a difference in the support of any coping ability. A meticulous follow-up with constant progress would make the first patient more optimistic and motivated. There is always a risk for a postoperative local infection after a nerve injury with an open wound, with or without fractures, particularly if necrotic tissues are present such as after gunshot wounds [30]. In such cases it is not advisable to perform any nerve reconstruction, but to do a meticulous revision of the wound and later a nerve reconstruction depending on the medical condition of the patient. In both the present cases the condition of the wounds were considered clean enough to perform the nerve reconstruction procedure early. However, in the second case a limited local infection occurred and was successfully treated, but we do not interpret the local infection responsible for the poor outcome. There seems to be a low infection rate after immediate nailing of femoral shaft fractures if the condition of the wound is addressed properly [55].

**Figure 4:**
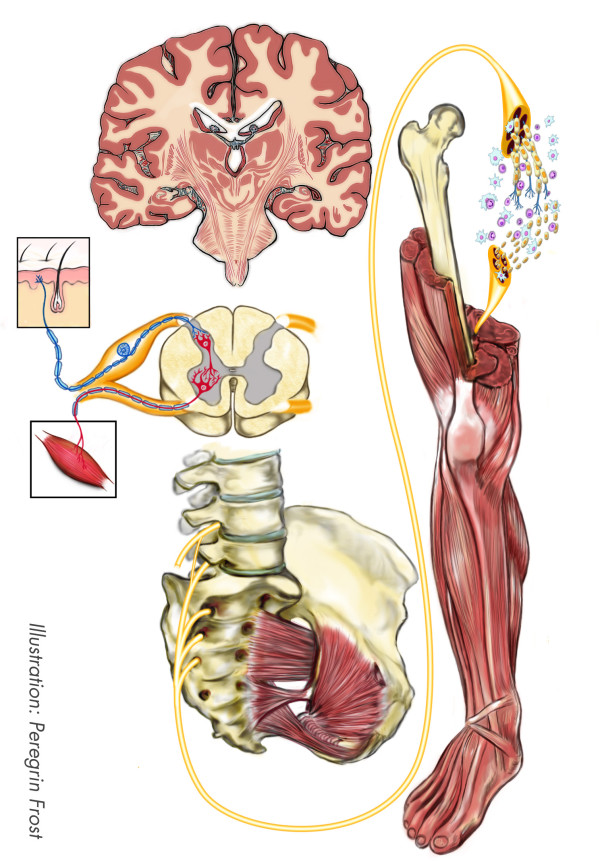
**Schematic drawing of a sciatic nerve at mid**-**thigh level with the potential factors that influence functional outcome extending from local signal transduction mechanisms in Schwann cells and neurons**, **secondary changes in the target areas**, **apoptosis of neurons in e**.**g**. **dorsal root ganglia**, **and reorganization at the cortical and subcortical levels.**

The two cases also had several things in common which allows an analysis of factors influencing the outcome equally and in a similar fashion (Figure 4). The injuries were rather similar in severity, i.e. laceration of the sciatic nerve with a nerve defect. Such injuries have a negative influence on the outcome [21]. This has been observed clinically and in experimental studies. Age is the factor most commonly accepted to influence outcome (18). Both patients presented here were adults with an age where the prognosis of a peripheral nerve injury is substantially worse than in children. The patients’ age is not in their favor, but equally against both of them.

Timing is of great importance due to the changes in both neuron and Schwann cells described above. In the present cases, the injury to the nerve was apparent either with presentation or soon after. Repair and reconstruction was performed within three days. Repair within this timeframe is referred to as delayed primary repair and reconstruction, a strategy generating as good results as can be expected with this kind of injuries [6].

The graft used for reconstruction in both cases was the sural nerve ipsilateral to the injury. The grafts had been denervated for two and three days, respectively, at a time point where the pre-degeneration was initiated in the ipsilateral sural nerve, i.e. a pre-degenerated nerve graft, which can be expected to promote axonal growth over the transplant [40]. In rat sciatic nerve models it is possible to use pre-degeneration on the sciatic nerve contra-lateral to the planned injury and use this as a graft. In humans, this is not easily arranged and the use of an autograft harvested a few days after injury is likely to be one of few strategies, which benefit from the effects of pre-degeneration.

Misdirection results in loss of hind leg functionality due to the simultaneous activation of opponent muscle groups [44]. This loss of functionality does not appear to be as relevant in humans, as the first patient regained good function in his leg, although he had some difficulties to selectively activate his toe flexor muscles properly at the last follow up, but this did not impaired his function. Locomotion studied in rats is an automated action and has less impact on human behavior. Also, with slower reinnervation rate and longer distances to overcome, muscle function is regained a little at a time with time to rehabilitate function gradually.

A better understanding of the biological mechanisms of peripheral nerve regeneration will hopefully improve the care of patients (Figure 4). Research in the rat model has shown that several factors can influence nerve regeneration and the outcome in patients. For example, the importance of timing of reconstruction and repair for outcome has been clearly shown in the rat sciatic nerve model. The conclusions from the experimental research have prompted the rapid reoperation and nerve repair in the cases presented here. However, repair and reconstruction is not all.

Cognitive capacity and coping strategies is an area where more research is needed. In this study, there is some indication to the importance of these factors. It is possible that the use of a rat model has limitations here.

Using fMRI, we demonstrated a normal contralateral activation in the S1 following cutaneous stimulation of the uninjured foot. Stimulation of the injured foot did not show any cortical activation in any of the subjects. This could be expected in the subjects lacking sensibility in the foot. However, the other subject had some sensibility in the foot. Even though a subject may perceive the stimulation, it is not certain that such cutaneous stimulation may be captured using fMRI. The hemodynamic response is highly individual and some individuals may have a more subtle response leading to statistics below the threshold of the fMRI analysis.

A strong correlation between DRG volume and the number of sensory neurons have been described [56]. Furthermore, the volume of DRG, quantified by MRI or by morphology, has been shown to correlate closely with the number of sensory neurons after a rat sciatic nerve injury. MR imaging of the human dorsal root ganglia has been previously described [57-59]. Normally, the DRG lie obliquely in the superolateral portion of the lumbar intervertebral foramen; thus, neither standard cross-sectional nor coronal imaging provides a view allowing for a comprehensive analysis of the DRG. Here, we measured the cross sectional area of the DRG on a reconstructed image through the largest portion of the ganglion and perpendicular to the length axis of the spinal nerve. We believe that this produce provides the most correct values. Previous studies on the rat sciatic nerve injury model have described volume reduction in the DRG following sciatic nerve injury [18]. To our knowledge, no previous studies exist that have used MRI to show any reduction in size or volume of DRG following a sciatic nerve injury in humans. Here, we could not show any differences in the size of the DRG following sciatic nerve and reconstruction. This could be due to several reasons. First of all, the effect on the DRG in terms of volume reduction following a sciatic nerve injury is not known in detail. The DRG at levels L4-S3 do not only support the sciatic nerve and volume loss, due to degeneration, might partly be prevented by activated glial cells and endoneurial macrophages that are presented in DRG for various reasons. Individual variability in formation and size of the ganglia at different levels can not been taken into account when studying only two subjects. Furthermore, although patients were examined with high resolution MR, artefacts and partial volume effects cannot be avoided due to still limited image resolution. Another possibility is that there is no volume loss in the DRG that supply the sciatic nerve in humans. In the future, further advanced neuroimaging techniques may be of great importance offering an opportunity to better understand the biology behind to regeneration process following a sciatic nerve injury. These techniques may also offer a possibility to monitor recovery of the nerve function and even the axonal outgrowth following injury and reconstruction.

The comparison between clinical research on patients and the results from studies on animals has the benefit of being translational in the most literal meaning of the word. The truly interesting part of any research conducted in the field of medicine must be the question: How does this affect the patient? However, the method of case reports has its limitations, but can be useful if the numbers of cases are rare and if they may generate new hypotheses. They may also add information and some clinical experience, which should be shared in the medical literature. Another methodological problem is the potential bias in the selection of articles for the background. The structured searched in the Pubmed database is designed to minimize this, but since only one person took part in the selection some bias is likely.

## Conclusions

The present paper, based on two cases with different outcome, reviews points that may be raised in decision making of nerve repair and reconstruction where experimental data can be useful to predict outcome in patients. A number of factors influence outcome of repair and reconstruction of nerve injuries, illustrated here in the two patients with injuries of the sciatic nerve, even if the factors behind the poor result in the second case are obvious. The rat sciatic nerve injury model is useful in identifying and studying factors that influence outcome after peripheral nerve repair and reconstruction, although such a model cannot completely mimic the dilemmas in the clinical situation. Results from experimental studies can be translated to clinical practice, although with caution and discernment as well as be related to clinical experience, and used in repair and reconstruction of nerve injuries in humans. However, some factors, such as cognitive capacity and coping, are difficult to study in rat models. Future research has to find and develop new paths and techniques to study the changes in the central nervous system after injury and develop strategies to utilize brain plasticity during rehabilitation.

## Abbreviations

DRG: Dorsal root ganglion; MRI: Magnetic Resonance imaging; fMRI: Functional Magnetic Resonance Imaging.

## Competing interests

The authors have no disclosures related to the present cases.

## Authors’ contributions

AM has, initially as a student project, written, together with the co-authors, the manuscript. LD has operated one of the cases and clinically followed up both cases. ABN, IBB and PM have performed the MRI and fMRI. GA has done the follow up with electrophysiology. All authors have read and approved the manuscript.
